# Lumbar functional evaluation of pelvic bone sarcomas after surgical resection and spinal pelvic fixation: A clinical study of 304 cases

**DOI:** 10.1002/cam4.7282

**Published:** 2024-05-31

**Authors:** Qianyu Shi, Wei Guo, Tao Ji, Xiaodong Tang

**Affiliations:** ^1^ Department of Musculoskeletal Tumor People's Hospital, Peking University Beijing China

**Keywords:** bone sarcomas, functional outcomes, lumbar functional index, pelvis, spinal pelvic fixation

## Abstract

**Aims:**

We endeavored to introduce a novel scoring system (Lumbar Functional Index, LFI) capable of evaluating lumbar function in pelvic bone sarcoma patients who underwent surgical resection and spinal pelvic fixation, while simultaneously identifying the incidence, outcomes, and risk factors of lumbar function impairment among these populations.

**Patients and Methods:**

A cohort of 304 primary bone sarcoma patients were recruited. The LFI was created based on the Oswestry Dysfunction Index (ODI) and Japanese Orthopaedic Association (JOA) scores. Lumbar function impairment was defined as LFI score ≥ 18 points, which was identified as high LFI. Demographic data, clinical characteristics, and oncological outcomes were analyzed.

**Results:**

The cohort included chondrosarcoma (39.8%), osteosarcoma (29.9%), Ewing sarcoma (8.6%), bone‐derived undifferentiated pleomorphic sarcoma (7.2%), giant cell tumor of bone (7.2%), chordoma (2.3%), and other bone sarcomas (5.0%). The LFI score exhibited significant negative correlation with common scoring systems of bone sarcoma. The incidence of high LFI was 23.0%. Patients with high LFI demonstrated a higher prevalence of type I + II + III + IV pelvic tumor, more sacrificed nerve roots and bilateral lumbar spine fixation during surgery, while lower percentage of R0 resection and local control of pelvic tumor. Decreased median overall survival (30 vs. 52 months, *p* < 0.001) and recurrence‐free survival (14 vs. 24 months, *p* < 0.001) time were observed in these patients. Type I + II + III + IV pelvic tumor and sacrificed nerve roots≥2 were identified as risk factors for high LFI, while R0 resection and local control were identified as protective factors.

**Conclusion:**

The LFI scoring system exhibited a significant negative correlation to current scoring systems. High LFI patients had worse prognosis and distinct characteristics. The risk factors of high LFI included type I + II + III + IV pelvic tumor and sacrificed nerve roots≥2, and the protective factors included R0 resection and local control.

## 1 INTRODUCTION

The prognosis of pelvic bone sarcomas is notably inferior when compared to those occurring in extremities.^1^, ^2^, ^3^, ^4^, ^5^ Surgical excision is the principal therapeutic intervention for pelvic bone sarcomas, with R0 resection representing a potentially curative option capable of significantly enhancing prognosis.^6^ However, the technical challenge of achieving R0 resection is compounded by the complex anatomic relationships with vital organs, major vessels, and nerves in the pelvic region.^6^, ^7^, ^8^

The utilization of spinal pelvic fixation (SPF) has been adopted in surgical reconstruction procedures following excision, with the intention of providing stabilization to the lumbosacral region.^9^ The adequacy and efficacy of SPF are critical factors that contribute significantly to the overall stability of the spinal column and postoperative lumbar function of patients. Furthermore, the degree of postoperative lumbar function is closely associated with patient quality of life and surgical satisfaction, underscoring the need for optimal implementation of SPF.

The dearth of information regarding postoperative lumbar function among patients with pelvic bone sarcomas who undergo surgical excision and spinal pelvic fixation (SPF) necessitates further research in this area. Additionally, current lumbar function scoring systems such as the Oswestry Dysfunction Index (ODI)^10^ and Japanese Orthopaedic Association (JOA)^11^, ^12^ score primarily target benign dysfunctional disorders such as lumbar spinal stenosis, spinal disc herniation, cervical spondylotic myelopathy, and etc. These scoring systems should be modified to match the extensive surgical resection and trauma associated with the procedure in pelvic sarcoma patients. Furthermore, it has been observed that the assessment tools designed to evaluate functional outcomes in individuals affected by bone sarcoma, such as the Patient Reported Outcomes Measurement Information System (PROMIS),^13^ Toronto Extremity Salvage Score (TESS),^14^ and Musculoskeletal Tumor Society (MSTS) scoring system,^15^ are designed to assess the general functional outcomes rather than a specific aspect of functional outcomes (for example, lumbar functional outcome). In this study, we proposed a novel scoring system (Lumbar Functional Index, LFI) for evaluating lumbar function among patients with pelvic sarcomas who underwent surgical excision and SPF. The LFI scoring system was proposed based on the ODI and JOA score and examined for validity via comparing the correlation between the LFI and three functional scores (the PROMIS, TESS, and MSTS). We conducted a retrospective analysis of lumbar function, clinical outcomes, and postoperative complications, and identified risk factors associated with poor lumbar function as indicated by high LFI scores. To the best of our knowledge, this is the largest cohort study focusing on postoperative lumbar function following SPF in pelvic bone sarcoma patients.

## 2 PATIENTS AND METHODS

A cohort of 304 patients diagnosed with primary bone sarcoma in the pelvic region and treated with surgical resection and SPF in our center between January 2017 and January 2022 were enrolled in the study, which received approval from the Medical Ethical Committees. Patients underwent surgical resection based on the pelvic tumor type according to the Enneking and Dunham classification,^16^ and the reconstruction options were briefly described as follows. In cases involving type I resection (involving the sacroiliac joint) and type I + IV resection, the reconstruction was accomplished using a screw‐rod system. For type I + II, I + II + III, I + II + IV, and I + II + III + IV resections, the reconstruction involved the utilization of screw‐rod hemipelvic endoprostheses. Exclusion criteria comprised of (1) bone tumor metastasized from other organs or tissues, (2) tumor arising from non‐bone tissues such as soft‐tissue and blood, (3) prior pelvic surgery or radiation therapy, and (4) incomplete data or loss to follow‐up. Demographic information, clinical characteristics, and prognostic data were collected for analysis.

### 2.1 The LFI scoring system

In this study, we proposed a scoring system named LFI, designed in a questionnaire format based on the ODI^10^ and JOA score.^12^ The LFI was modified to account for the significant trauma resulting from surgery in some specific items such as bladder function, walking, and social life. The LFI consists of nine major items including pain, sleeping, personal care, bladder function, bowel function, walking, sitting, lifting, and social life (Table 1). Each major item is graded on a scale of 0 to 5 according to the symptom severity, resulting in a total score ranging from 0 to 45. A lower score indicates milder symptoms and better lumbar function. In each major item, a score of 2 or more is a cutoff value that represents an obviously abnormal function and may have a significant impact on the patient's quality of life. Consequently, the cutoff value of total score for defining high LFI score/poor lumbar function was established as 18 points.

**TABLE 1:** The lumbar functional index (LFI) after spinal pelvic fixation in pelvic bone sarcoma patients.

Sections score	
Pain	
I have no pain at all	0
I have tolerable pain with no need to take painkillers	1
I have to take non‐steroidal anti‐inflammatory drugs (NSAIDs) to relieve pain	2
I have to take weak opioid medications to relieve pain, sometimes combined with NSAIDs	3
I have to take strong opioid medications to relieve pain	4
Even strong opioid medications have no effect on the pain	5
Sleeping	
I am very satisfied with the quality of my sleep	0
I can achieve a good quality of sleep by relying on pills	1
My sleep duration is less than 6 h even if I take pills	2
My sleep duration is less than 4 h even if I take pills	3
My sleep duration is less than 2 h even if I take pills	4
Pain prevents me from falling asleep at all	5
Personal care	
I can take care of myself normally without causing extra pain	0
I can take care of myself normally but it causes extra pain	1
I cannot take care of myself normally due to the pain so I am slow and careful	2
I sometimes need others help to take care of myself	3
I often need others help to take care of myself	4
I always need others help and I cannot take care of myself at all	5
Bladder function	
I have normal bladder function	0
I have relatively normal bladder function but slightly different from others	1
I have mild bladder dysfunction	2
I have middle bladder dysfunction	3
I have severe bladder dysfunction	4
I have urinary retention and cannot control my bladder	5
Bowel function	
I have normal bowel function	0
I have relatively normal bowel function but slightly different from others	1
I have mild bowel dysfunction	2
I have middle bowel dysfunction	3
I have severe bowel dysfunction	4
I have fecal incontinence and cannot control defecation	5
Walking	
I can walk normally like other people, without the need for crutches or walkers	0
I need to use crutches/walkers to walk and I can walk 1000 m a time	1
I need to use crutches/walkers to walk and I can walk 500 m a time	2
I need to use crutches/walkers to walk and I can walk 100 m a time	3
I need to use crutches/walkers to walk and I can walk less than 100 m a time	4
I cannot walk at all even with crutches/walkers	5
Sitting	
I can sit in any chair as long as I like	0
I can sit in a comfortable chair as long as I like	1
Pain and soreness prevent me from sitting more than 1 h	2
Pain and soreness prevent me from sitting more than 30 min	3
Pain and soreness prevent me from sitting more than 10 min	4
Pain and soreness prevent me from sitting at all	5
Lifting	
I can lift heavy weights without extra pain	0
I can lift heavy weights but it causes extra pain	1
I cannot lift heavy weights off the floor but I can lift heavy weights off the table	2
I can lift medium weights off the table	3
I can lift only light weights off the table	4
I cannot lift or carry anything at all	5
Social life	
I have a normal social life without extra pain	0
I have a normal social life but it causes extra pain	1
Pain has slightly limited my social life	2
Pain has moderately limited my social life	3
Pain has severely limited my social life	4
I have no social life due to the pain	5
Total	45

### 2.2 Validity of the LFI scoring system

The LFI scoring system was validated by analyzing the correlation between the LFI and the PROMIS,^13^ TESS,^14^ and MSTS^15^ scoring systems. The PROMIS Global Short Form and the lower extremity MSTS score were used in this study. Spearman's rank correlation test was utilized for validation and a *p*‐value<0.05 was considered statistically significant. Data related to the LFI, PROMIS, TESS, and MSTS scores were collected retrospectively as follows. The provider interviewed the patient for the questions and the scores were reported by the provider.

### 2.3 Follow‐up

The patients were subject to regular follow‐ups, at 3‐month intervals for the initial 2 years after undergoing surgery, and subsequently at 6‐month intervals until 5 years after surgery. During each follow‐up visit, the patients underwent X‐ray imaging and CT or MRI, if necessary, to evaluate the surgical region. Chest X‐ray or CT scan was performed to identify any lung metastases, while whole‐body bone scan was conducted to detect bone metastases. The data pertaining to the LFI score was collected via a search of the medical records and telephone calls conducted at the final follow‐up, which occurred in September 2023. The mean follow‐up was 40.8 ± 15.2 months (12.0 to 66.0).

### 2.4 Statistical analysis

Statistical analyses were conducted using GraphPad Prism 8.0. Receiver operating characteristic (ROC) curve analysis was employed to determine a cutoff value for continuous variables. Student's *t* test was utilized for normally distributed continuous data, while Mann–Whitney test was utilized for non‐normally distributed continuous data. Categorical variables were assessed using *X* ^*2*^ or Fisher's exact tests. Variables with a *p* value of <0.1 in the univariate analysis were entered into a binary logistic regression model for multivariate analysis. Survival analysis was performed using Kaplan–Meier curves and the log‐rank test. A *p*‐value<0.05 was considered statistically significant.

## 3 RESULTS

### 3.1 Demographic data, clinical characteristics, and validation of the LFI scoring system

**FIGURE 1:**
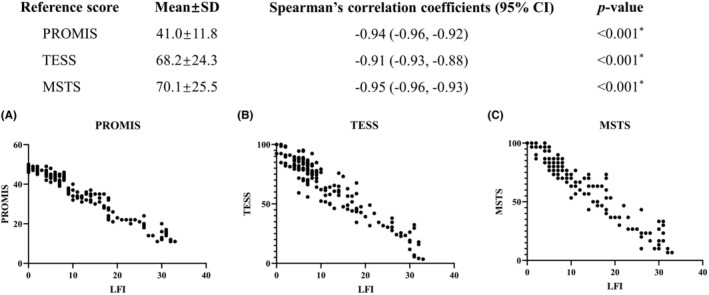
Scatterplot of the distribution of the LFI score and reference scores. (A) The LFI and the PROMIS scores. (B) The LFI and the TESS scores. (C) The LFI and the MSTS scores. All data exhibited a significant negative correlation between the LFI score and reference scores. *p‐value <0.05.

The present study enrolled a total of 304 participants, of which 178 (58.5%) were males and 126 (41.5%) were females. The mean age of the study population was 41.5 ± 16.2 (mean ± SD) years, as demonstrated in Table 2. The average duration between the onset of symptoms and medical consultation was 9.2 ± 15.2 months. The most prevalent types of sarcomas detected in this study were chondrosarcoma (39.8%), osteosarcoma (29.9%), and Ewing sarcoma (8.6%). The average tumor size was found to be 112.9 ± 44.9 mm. With regard to lumbar segment involvement, L4 (96.1%) was the most frequently involved for fixation, followed by L3 (67.4%), L5 (50.3%), L2 (8.2%), and L1 (2.0%). Of note, the majority of patients underwent lumbar fixation of two segments (75.3%) and bilateral lumbar fixation (85.9%) was performed in most cases. The LFI scores exhibited a significant negative correlation with the PROMIS, TESS, and MSTS, with a Spearman's correlation coefficient of −0.94 (95% CI [−0.96, −0.92], *p* < 0.001), −0.91 (95% CI [−0.93, −0.88], *p* < 0.001), and − 0.95 (95% CI [−0.96, −0.93], *p* < 0.001), respectively (Figure 1). Patients with high LFI exhibited a higher prevalence of type I + II + III + IV pelvic tumor (22.9% vs. 1.3%, *p* < 0.001, Table S1) and preoperative metastatic disease (47.1% vs. 29.5%, *p* < 0.05), advanced age (46.7 ± 14.5 vs. 38.0 ± 16.2, *p* < 0.05), more sacrificed nerve roots (2.51 ± 1.75 vs. 0.49 ± 1.23, *p* < 0.001, Table 3) and bilateral lumbar spine fixation (94.3% vs. 83.3%, *p* < 0.05), while lower percentage of R0 resection (70.0% vs. 93.6%, *p* < 0.001) and local control (31.4% vs. 71.4%, *p* < 0.001) of pelvic tumor compared to their low LFI counterparts.

**TABLE 2:** Demographic and clinical characteristics of 304 cases of pelvic bone sarcomas.

Variables	Value
Sex (no. [%])	
Male	178 (58.5)
Female	126 (41.5)
Age^a^ (year)	41.5 ± 16.2
Height^a^ (cm)	165.8 ± 10.0
Weight^a^ (kg)	61.7 ± 12.0
Body mass index^a^ (kg/m^2^)	22.4 ± 3.9
Time from symptom onset to presentation^a^ (month)	9.2 ± 15.2
Pathological diagnosis (no. [%])	
Chondrosarcoma	121 (39.8)
Grade I	5 (4.1)
Grade 1‐II	22 (18.2)
Grade II	50 (41.3)
Grade II‐III	4 (3.3)
Grade III	1 (1.0)
Dedifferentiated	25 (207)
Clear cell	1 (1.0)
Mesenchymal	13 (10.7)
Osteosarcoma	91 (29.9)
Chondroblastic	30 (33.0)
Osteoblastic	16 (17.6)
Telangiectatic	2 (2.2)
Fibroblastic	3 (3.3)
Small cell	3 (3.3)
Well‐differentiated	6 (6.6)
Undifferentiated pleomorphic sarcoma‐like	1 (1.1)
Epithelioid	1 (1.1)
Not otherwise specified	29 (31.9)
Ewing sarcoma	26 (8.6)
Chordoma	7 (2.3)
Bone‐derived undifferentiated pleomorphic sarcoma	22 (7.2)
Giant cell tumor of bone	22 (7.2)
Other	15 (4.9)
Stage (no. [%])	
Localized	202 (66.4)
Metastatic^b^	102 (33.6)
Type of pelvic tumor^c^ (no. [%])	
I	21 (6.9)
I + II	53 (17.4)
I + II + III	30 (9.9)
I + II + III + proximal part of femur	4 (1.3)
I + IV	123 (40.5)
I + II + IV	54 (17.8)
I + II + III + IV	13 (4.3)
I + II + III + IV + proximal part of femur	6 (2.0)
Tumor size^a^ (mm)	112.9 ± 44.9
Chemotherapy	174 (57.2)
Radiotherapy	32 (10.5)
Number of sacrificed nerve roots	1.0 ± 1.6
Fixed segments of the lumbar spine (no. [%])	
L1	6 (2.0)
L2	25 (8.2)
L3	205 (67.4)
L4	292 (96.1)
L5	153 (50.3)
Number of fixed segments of the lumbar spine (no. [%])	
1	4 (1.3)
2	229 (75.3)
3	66 (21.7)
4	5 (1.6)
5	0 (0)
Range of lumbar spine fixation (no. [%])	
Unilateral	43 (14.1)
Bilateral	261 (85.9)

^a^ The values are given as the mean and standard deviation.

^b^ The metastatic disease in the cohort were all lung metastasis and operable. The indication for surgery in patients with metastatic disease is metastatic lesions that are surgically excisable.

^c^ According to the Enneking and Dunham classification. Type I pelvic tumor not inclusive of the sacroiliac joint was excluded.

**TABLE 3:** Comparison of demographic and clinical characteristics between patients with low LFI and high LFI.

Variables	Low LFI (*n* = 234)	High LFI (*n* = 70)	*p* Value
Stage (no. [%])			
Localized	165 (70.5)	37 (52.9)	0.009*
Metastatic	69 (29.5)	33 (47.1)	
Tumor size^a^ (mm)	102.9 ± 39.1	115.5 ± 39.8	0.080
Pathological diagnosis (no. [%])			
Chondrosarcoma	92 (39.3)	29 (41.4)	0.592
Osteosarcoma	66 (28.2)	25 (35.7)	
Ewing sarcoma	23 (9.8)	3 (4.3)	
Chordoma	5 (2.1)	2 (2.9)	
Bone‐derived undifferentiated pleomorphic sarcoma	18 (7.7)	4 (5.7)	
Giant cell tumor of bone	19 (8.1)	3 (4.3)	
Other	11 (4.7)	4 (5.7)	
Age^a^ (year)	38.0 ± 16.2	46.7 ± 14.5	0.002*
Sex (no. [%])			
Male	134 (57.3)	44 (62.9)	0.490
Female	100 (42.7)	26 (37.1)	
Chemotherapy (no. [%])			
No	101 (43.2)	29 (41.4)	0.891
Yes	133 (56.8)	41 (58.6)	
Radiotherapy (no. [%])			
No	210 (89.7)	62 (88.6)	0.825
Yes	24 (10.3)	8 (11.4)	
Postoperative complications^b^ (no. [%])			
No	172 (73.5)	48 (68.6)	0.448
Yes	62 (26.5)	22 (31.4)	
Postoperative complications that needed surgical treatment (no. [%])			
No	193 (82.5)	51 (72.9)	0.087
Yes	41 (17.5)	19 (27.1)	
Number of sacrificed nerve roots^a^	0.49 ± 1.23	2.51 ± 1.75	<0.001*
Number of fixed segments of the lumbar spine^a^	2.2 ± 0.5	2.2 ± 0.5	0.862
Fixed segments of the lumbar spine (no. [%])^c^			
L1	4 (1.7)	2 (2.9)	0.676
L2	19 (8.1)	6 (8.6)	
L3	154 (65.8)	51 (72.9)	
L4	222 (94.9)	70 (100)	
L5	124 (53.0)	29 (41.4)	
Range of lumbar spine fixation (no. [%])			
Unilateral	39 (16.7)	4 (5.7)	0.019*
Bilateral	195 (83.3)	66 (94.3)	

^a^ The values are given as the mean and standard deviation.

^b^ Postoperative complications included wound infection, poor wound healing, nerve compression, and loosening/fractures/dislocation of internal fixation implants.

^c^ Some patients had more than one segment of lumbar spine fixation.

* *p* Value <0.05.

### 3.2 Oncological outcomes

**FIGURE 2:**
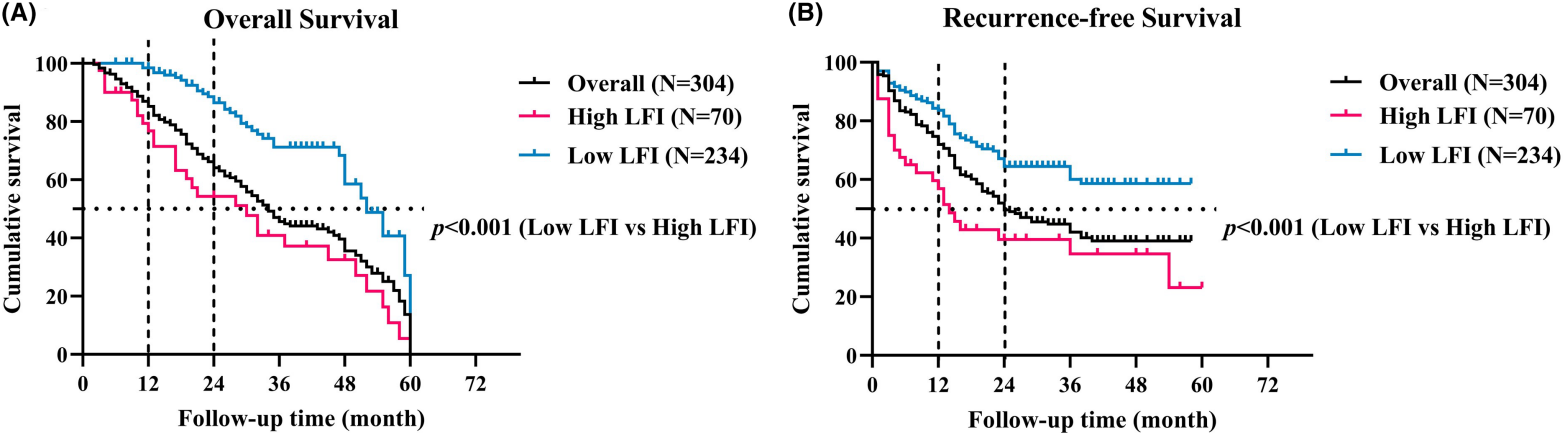
Kaplan–Meier curves with the log‐rank test of the overall (A) and recurrence‐free (B) survival of patients with low LFI compared with high LFI.

The median overall survival (OS) and recurrence‐free survival (RFS) times for the cohort were 34 months and 24 months, respectively (Figure 2). A statistically significant association was observed between high LFI and worse oncological outcomes, as evidenced by lower median OS and RFS times. Specifically, patients with high LFI had a median OS time of 30 months compared to 52 months for those with low LFI (*p* < 0.001) and a median RFS time of 14 months compared to 24 months for those with low LFI (*p* < 0.001).

### 3.3 Prevalence of high LFI and score details of high LFI patients

**FIGURE 3:**
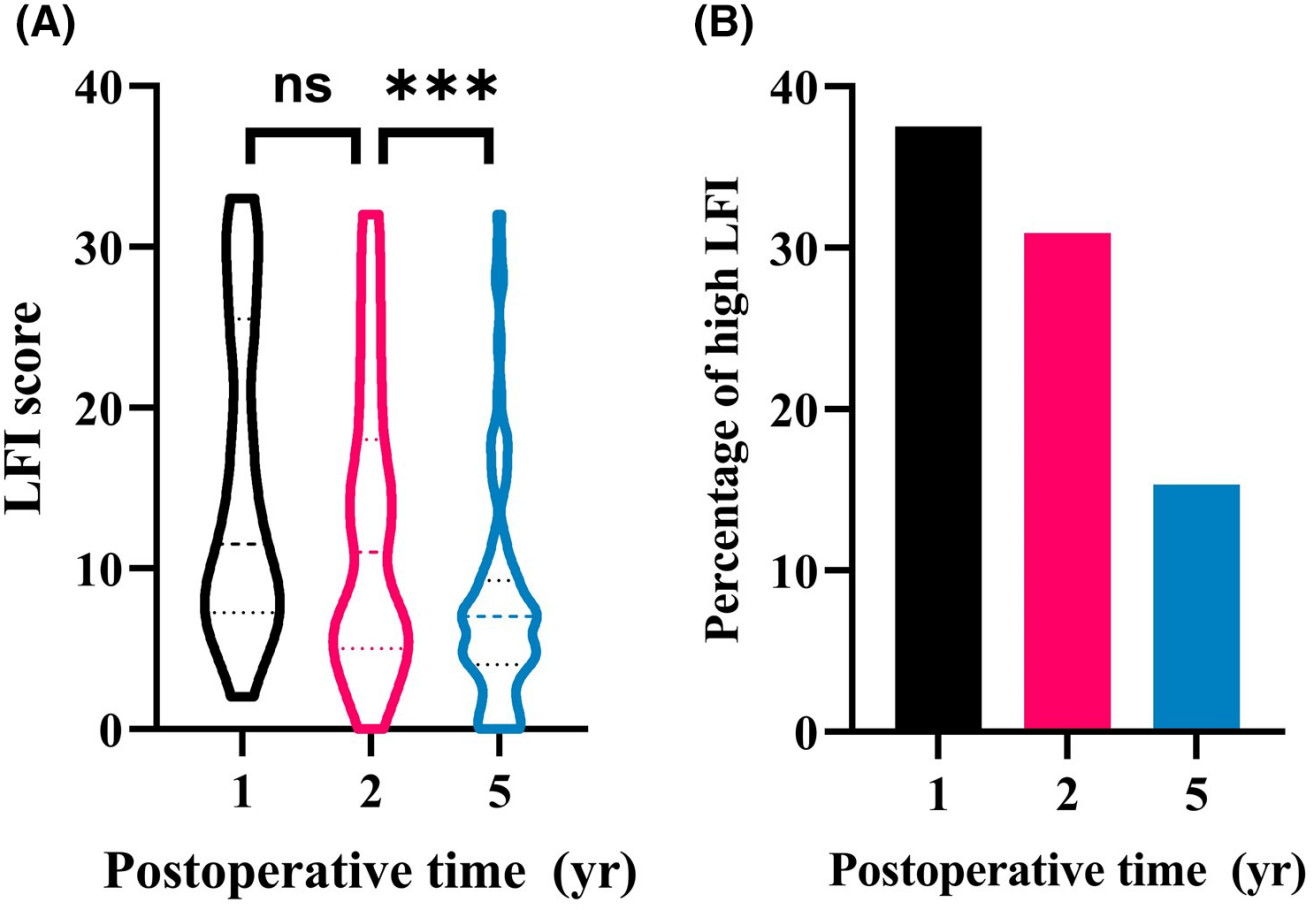
Relationship of the LFI score for each patient (A) and proportion of high LFI among the whole cohort (B) over time. ***, *p*‐value<0.001.

In the investigated cohort, 70 cases (23.0%) were identified as having high LFI, with LFI scores ranging from 18 to 33. The LFI started to decrease 2 years after surgery (Figure 3A) and the proportion of patients with high LFI were 37.5%, 30.9%, and 15.3% for the 1‐year, 2‐year, and 5‐year time intervals, respectively (Figure 3B). Among the individuals with high LFI, the major items of social life, personal care, and walking had the highest scores, with means of 3.85 ± 0.85, 3.67 ± 0.82, and 3.61 ± 1.02, respectively, as depicted in Figure S1. In contrast, bladder function, bowel function, and sleeping demonstrated mean scores below 2, indicating relatively normal function.

### 3.4 Risk factors for high LFI

**FIGURE 4:**
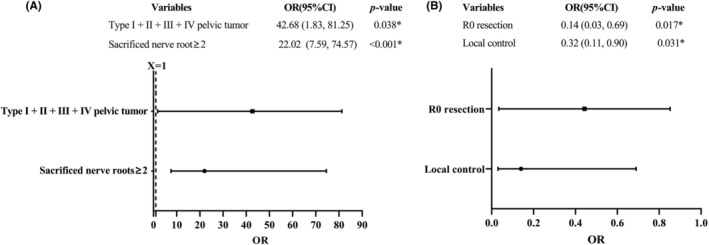
Multivariate analysis of logistic regression of high LFI. Type of pelvic tumor and sacrificed nerve roots≥2 were risk factors for patients having high LFI (A), while protective factors included R0 resection and local control (B). * means *p* value<0.05.

In the present study, a univariate analysis was performed to assess the association of multiple parameters with high LFI incidence within the cohort. The results of the analysis revealed that postoperative time (defined as the time interval from the surgery to the time point of follow‐up), tumor size≥116 mm, age at diagnosis, sacrificed nerve roots≥2, type I + II + III + IV pelvic tumor, R0 resection, and local control were significantly associated with high LFI occurrence (Table S2). Furthermore, multivariate analysis was conducted to evaluate the independent effects of these variables on high LFI incidence, wherein type I + II + III + IV pelvic tumor and sacrificed nerve roots≥2 were found to be significantly associated with a higher rate of high LFI (Figure 4A) while R0 resection and local control were identified as protective factors (Figure 4B).

## 4 DISCUSSION

Radical surgical resection has been the major treatment option for patients with primary pelvic bone sarcomas.^17^, ^18^, ^19^, ^20^ The assessment of the therapeutic effectiveness of surgical resection can be divided into two major aspects^21^, ^22^, ^23^: one is the oncological outcomes that is associated with patient survival, the other is the functional outcomes influencing patient quality of life. The MSTS and TESS scores designed for evaluation of postoperative functional outcomes are the most widely used scores for musculoskeletal tumor.^24^ The PROMIS is a patient reported outcome measure that has been proved to have high reliability and validity for sacral tumor patients.^13^ However, evaluation of lumbar function is inadequate for pelvic bone sarcoma patients who have undergone surgical resection and SPF. Commonly used evaluation of lumbar function such as the ODI and JOA scores mainly focus on benign diseases.^25^, ^26^ The scoring criteria for postoperative pelvic tumor patients should be modified accordingly since the relatively worse function caused by extensive surgical resection. In this study, we proposed the LFI scoring system based on the ODI and JOA scores. To our knowledge, this is the first and largest retrospective study of postoperative lumbar functional evaluation of primary bone sarcoma patients.

In addition to some categories that overlap with the MSTS/TESS/PROMIS, the LFI includes categories that reflect lumbar function specifically and has modified scoring criteria because of the worse lumbar function in the cohort. Consequently, the LFI scoring system specifically evaluates the lumbar function of patients with postoperative pelvic bone sarcomas in comparison with previous scoring systems, and is more suitable for these patients in comparison with ODI and JOA because of the extensive trauma caused by the surgery. To verify its validity, the LFI scoring system proposed in this study was assessed by Spearman's rank correlation test, which exhibited a significant correlation with the PROMIS, TESS, and MSTS scores. The score of LFI in the cohort ranged from 0 to 33 and the incident rate of high LFI was 23.0%. Interestingly, lumbar function of studied cohort ameliorated over time with the percentage of high LFI patients dropping from 37.5% to 15.3% during the first 5 years after surgery. The score distribution of high LFI patients exhibited discrepancy among different major items. Social life, personal care, and walking were the top three items that had the highest score while bladder function, sleeping, and bowel function had mean scores less than 2 points. Among the top three items, walking was the basic item that may set the tone of social life as well as personal care,^27^, ^28^ since patients who had trouble with walking would need extra help in self‐care and be more inclined to decrease the social frequency. The mean scores lower than 2 points demonstrated that high LFI patients had a relatively normal function in bladder control, defecation, and sleeping. Bladder and bowel dysfunction of postoperative pelvis bone sarcoma patients are mainly related to the injury of nerves caused by surgical procedures.^29^ Insomnia correlates with anxiety and pain closely and could be improved by sleeping pills and analgesics.^30^, ^31^ Therefore, high LFI patients with bad lumbar function could have a relatively normal score of bladder function, bowel function, and sleeping.

Patients with high LFI were prone to have older age, bilateral lumbar spine fixation, tumor metastases, type I + II + III + IV pelvic tumor, and sacrificed nerve roots, while R0 resection and local control were less common. The oncological outcomes including OR and RFS times for patients with high LFI were poorer, which may be due to the higher ratio of tumor metastasis in these patients. Identification of risk factors for high LFI is helpful for prediction the postoperative lumbar function in this population. Postoperative time interval was of importance in postoperative lumbar function improvement, although the interval could be very long since the 2‐year high LFI rate were similar to 1‐year high LFI rate in this cohort. The similarity of the 1‐year and 2‐year high LFI rate indicated the slow postoperative rehabilitation process due to the huge trauma. Nerve root sacrifice related to postoperative function directly and was a major risk factor of high LFI. Extensive excision and huge trauma (Figure S2) caused by type I + II + III + IV pelvic tumor resection was another risk factor of high LFI. In contrast, R0 resection and local control could avoid local recurrence that may lead to worsening function.

There are several limitations of the study. Firstly, it was a single‐center study and its retrospective nature as well as extended time frame increases the risk of selective and recall bias because data of the scores was collected retrospectively. The center is one of the largest in China for the treatment of musculoskeletal tumors, which results in a higher prevalence of more severe disease conditions among patients enrolled in this study. A prospective multicenter study is needed for further investigation. Secondly, the evaluation of lumbar function in this study was reliant on subjective assessment, with a lack of objective data on the patients' lumbar range of motion. Collection of data on the patients' lumbar range of motion at each follow‐up visit is conducive to acquire objective metrics of lumbar function. Third, the LFI scoring system proposed in this study requires verification of its reliability and further validation in additional cohorts. Future studies verifying the reliability and validity of the LFI scoring system may consider the following aspects: (1) Assess the reproducibility of the LFI scoring system using the test–retest reliability with intraclass correlation coefficient; (2) Prospectively recruit a cohort of primary bone sarcoma patients, verify the correlation of the LFI scores with common scoring systems of bone sarcoma in the cohort at different time points; (3) Analyze the ceiling and floor effects of each category and the total score of the LFI scoring system.

## 5 CONCLUSIONS

In summary, we proposed a novel scoring system for evaluation of postoperative lumbar function among pelvic sarcomas patients who underwent surgical excision and SPF. The LFI score exhibited a significant negative correlation to patient's functional outcome. Patients with high LFI had distinct characteristics and worse oncological outcomes. Risk factors for high LFI included type I + II + III + IV pelvic tumor and sacrificed nerve roots≥2, and the protective factors included R0 resection and local control. Further investigations should be conducted to examined the reliability of the LFI scoring system and discover underlying factors that could predict postoperative lumbar function.

## AUTHOR CONTRIBUTIONS

**Qianyu Shi:** Data curation (equal); formal analysis (equal); investigation (equal); methodology (equal). **Wei Guo:** Conceptualization (equal); funding acquisition (equal). **Tao Ji:** Data curation (equal); methodology (equal); writing – review and editing (equal). **Xiaodong Tang:** Methodology (equal); project administration (equal); resources (equal); supervision (equal).

## FUNDING INFORMATION

National Natural Science Foundation of China (82072970).

## CONFLICT OF INTEREST STATEMENT

The authors declare no potential conflict of interest.

## ETHICS STATEMENT

The study has received approval from the Medical Ethical Committees of Peking University People's Hospital.

## PATIENT CONSENT STATEMENT

Informed consent was obtained for patients enrolled in this study.

## Supporting information


Figure S1.

Figure S2.

Tables S1–S2.

## Data Availability

The original data of this study is available upon reasonable request from the corresponding author.
