# Supplementary material for: Lumbar functional evaluation of pelvic bone sarcomas after surgical resection and spinal pelvic fixation: A clinical study of 304 cases
Source: Cancer Med. 2024 May 31;13(11):e7282. doi: 10.1002/cam4.7282 (PMC11140840; doi:10.1002/cam4.7282)
Supplement: Supplementary file 1 — Figure S1. [file CAM4-13-e7282-s001.zip › Figure S1 caption.docx]

**Figure S1: Score distribution of each major item in high LFI patients.** Social life, personal care and walking were the top 3 items that had the highest scores among eight major items, while bladder function, bowel function and sleeping displayed an average score below 2, indicative of a relatively normal function in this patient cohort.
