# Supplementary figures and images for: Lumbar functional evaluation of pelvic bone sarcomas after surgical resection and spinal pelvic fixation: A clinical study of 304 cases
Source: Cancer Med. 2024 May 31;13(11):e7282. doi: 10.1002/cam4.7282 (PMC11140840; doi:10.1002/cam4.7282)

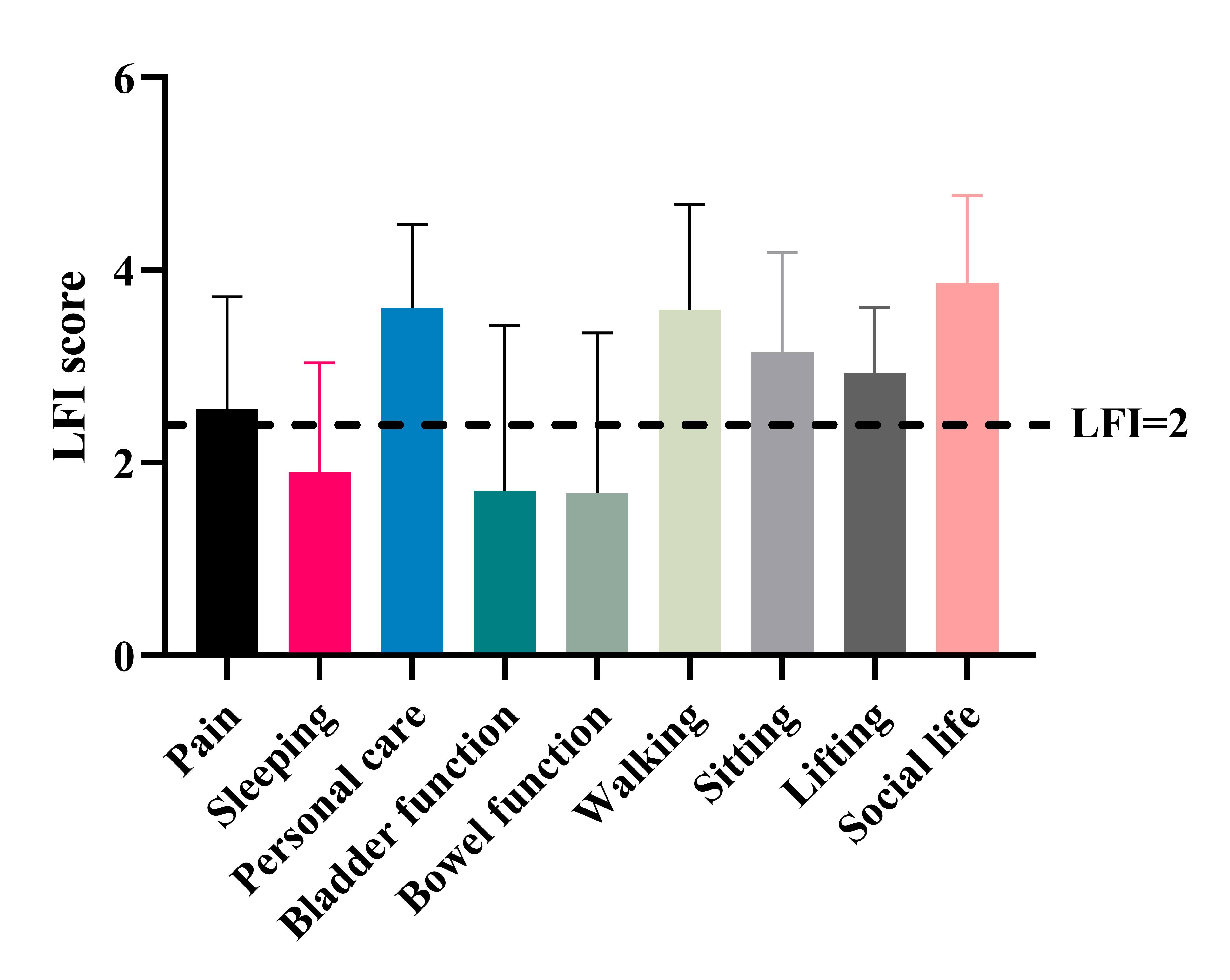

Supplement: Supplementary file 1 — Figure S1. [file CAM4-13-e7282-s001.zip › cam47282-sup-0001-Figure_S1.tif]

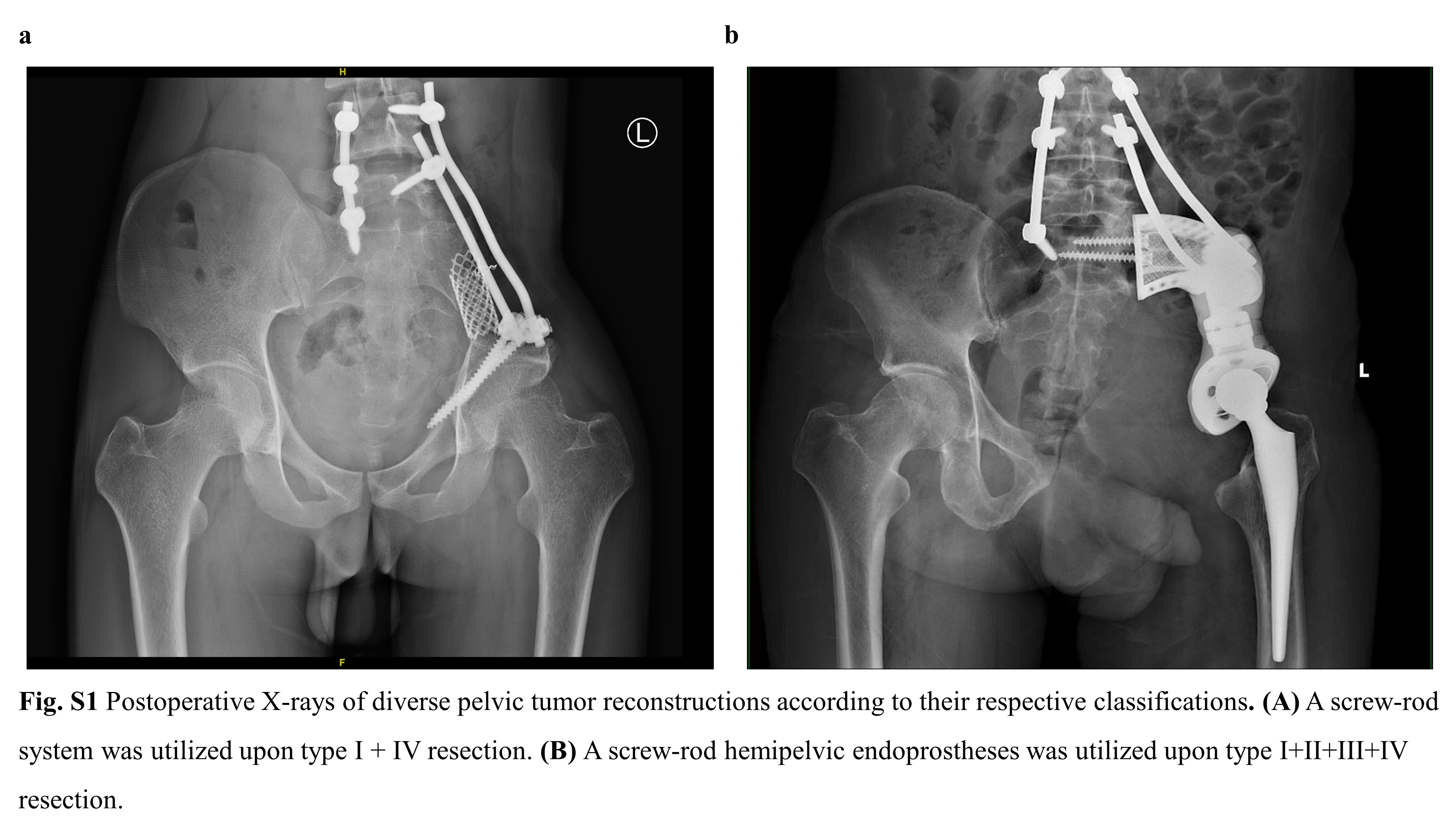

Supplement: Supplementary file 2 — Figure S2. [file CAM4-13-e7282-s003.zip › cam47282-sup-0002-Figure_S2.jpg]
