# Supplementary material for: Lumbar functional evaluation of pelvic bone sarcomas after surgical resection and spinal pelvic fixation: A clinical study of 304 cases
Source: Cancer Med. 2024 May 31;13(11):e7282. doi: 10.1002/cam4.7282 (PMC11140840; doi:10.1002/cam4.7282)
Supplement: Supplementary file 2 — Figure S2. [file CAM4-13-e7282-s003.zip › Figure S2 caption.docx]

**Figure S2: Postoperative X-rays of diverse pelvic tumor reconstructions according to their respective classifications.** (a) A screw-rod system was utilized upon type I + IV resection. (b) A screw-rod hemipelvic endoprostheses was utilized upon type I + II + III + IV resection.
