# Supplementary material for: Lumbar functional evaluation of pelvic bone sarcomas after surgical resection and spinal pelvic fixation: A clinical study of 304 cases
Source: Cancer Med. 2024 May 31;13(11):e7282. doi: 10.1002/cam4.7282 (PMC11140840; doi:10.1002/cam4.7282)
Supplement: Supplementary file 3 — Tables S1–S2. [file CAM4-13-e7282-s002.docx]

**Supplemental Table 1 Surgical procedures and outcomes in the cohort**

| **Varibales** | | **Low LFI**  **(n=234)** | **High LFI**  **(n=70)** | **Total**  **(n=304)** | ***p*-value§** |
| --- | --- | --- | --- | --- | --- |
| Type of pelvic tumor† | I  I + II  I + II + III  I + IV  I + II + IV  I + II + III + IV | 15 (6.4)  44 (18.8)  24 (10.3)  104 (44.4)  44 (18.8)  3 (1.3) | 6 (8.6)  9 (12.9)  10 (14.3)  19 (27.1)  10 (14.3)  16 (22.9) | 21 (6.9)  53 (17.4)  34 (11.2)  123 (40.5)  54 (17.8)  19 (6.2) | <0.001* |
| Reconstruction methods | screw-rod system  screw-rod hemipelvic endoprosthesis | 105 (44.9)  129 (55.1) | 29 (41.4)  41 (58.6) | 134 (44.1)  170 (55.9) | 0.681 |
| R0 resection  No  Yes | | 15 (0.4)  219 (93.6) | 21 (30.0)  49 (70.0) | 36 (11.8)  268 (88.2) | <0.001* |
| Local control  No  Yes | | 67 (28.6)  167 (71.4) | 48 (68.6)  22 (31.4) | 115 (37.8)  189 (62.2) | <0.001* |

**p*-value<0.05. §Comparison between the Low LFI and High LFI groups. † According to the Enneking and Dunham classification. I + II + III + proximal part of femur and I + II + III + IV + proximal part of femur were added to I + II + III and I + II + III + IV respectively due to the small sample size.

**Supplemental Table 2** Risk factors for high LFI in the cohort

| **Variables** | **Bivariate analysis** | | | **Multivariate analysis** | |
| --- | --- | --- | --- | --- | --- |
|  | OR | 95% CI | *p*-value | OR (95% CI) | *p*-value |
| Postoperative time (m) | 0.95 | 0.92, 0.98 | 0.002* | 0.97 (0.91, 1.03) | 0.377 |
| Stage | 0.25 | 0.15, 0.39 | 0.099 | 0.92 (0.31, 2.65) | 0.876 |
| Tumor size (mm) | 1.01 | 0.99, 1.02 | 0.064 |  |  |
| Tumor size≥116 mm† | 2.16 | 1.05, 4.48 | 0.036* | 0.83 (0.28, 2.39) | 0.734 |
| Pathological diagnosis | 0.96 | 0.35, 2.60 | 0.903 |  |  |
| Age | 1.04 | 1.02, 1.07 | 0.002* | 0.9997 (0.97, 1.03) | 0.985 |
| Sex | 1.33 | 0.64, 2.84 | 0.449 |  |  |
| Chemotherapy | 1.13 | 0.53, 2.45 | 0.787 |  |  |
| Radiotherapy | 1.26 | 0.61, 3.25 | 0.624 |  |  |
| Postoperative complications | 1.04 | 0.45, 2.26 | 0.928 |  |  |
| Postoperative complications that needed surgical treatment | 1.74 | 0.70, 4.13 | 0.393 |  |  |
| Number of sacrificed nerve roots | 2.09 | 1.64, 2.75 | <0.001* |  |  |
| Sacrificed nerve roots≥2† | 17.27 | 7.16, 44.88 | <0.001* | 22.02 (7.59, 74.57) | <0.001* |
| Number of fixed segments of the lumbar spine | 0.72 | 0.32, 1.49 | 0.407 |  |  |
| Range of lumbar spine fixation | 0.25 | 0.01, 1.30 | 0.184 |  |  |
| Type of pelvic tumor※ | 29.17 | 3.87, 64.5 | 0.004* | 42.68 (1.83, 81.25) | 0.038* |
| Reconstruction methods | 1.41 | 0.67, 3.06 | 0.439 |  |  |
| R0 resection | 0.14 | 0.04, 0.40 | 0.029* | 0.14 (0.03, 0.69) | 0.017* |
| Local control | 0.15 | 0.06, 0.33 | <0.001* | 0.32 (0.11, 0.90) | 0.031* |

† ROC curve analysis resulted in a tumor size of 116 mm (area under the curve [AUC]: 0.62, p < 0.05) and sacrificed nerve roots of 2 (AUC: 0.80, p < 0.001) as cutoff values. ※represents for Type I + II + III + IV pelvic tumor. **p*-value<0.05.
